# Women with a History of Childhood Maltreatment Exhibit more Activation in Association Areas Following Non-Traumatic Olfactory Stimuli: A fMRI Study

**DOI:** 10.1371/journal.pone.0009362

**Published:** 2010-02-22

**Authors:** Ilona Croy, Julia Schellong, Johannes Gerber, Peter Joraschky, Emilia Iannilli, Thomas Hummel

**Affiliations:** 1 Department of Psychosomatic Therapy, University of Dresden Medical School, Dresden, Germany; 2 Department of Otorhinolaryngology, University of Dresden Medical School, Dresden, Germany; 3 Department of Neuroradiology, University of Dresden Medical School, Dresden, Germany; James Cook University, Australia

## Abstract

**Background:**

The aim of this study was investigating how women with a history of childhood maltreatment (CM) process non-threatening and non-trauma related olfactory stimuli. The focus on olfactory perception is based on the overlap of brain areas often proposed to be affected in CM patients and the projection areas of the olfactory system, including the amygdala, orbitofrontal cortex, insula and hippocampus.

**Methods:**

Twelve women with CM and 10 controls participated in the study. All participants were, or have been, patients in a psychosomatic clinic. Participants underwent a fMRI investigation during olfactory stimulation with a neutral (coffee) and a pleasant (peach) odor. Furthermore, odor threshold and odor identification (Sniffin' Sticks) were tested.

**Principal Findings:**

Both groups showed normal activation in the olfactory projection areas. However, in the CM-group we found additionally enhanced activation in multiple, mainly neocortical, areas that are part of those involved in associative networks. These include the precentral frontal lobe, inferior and middle frontal structures, posterior parietal lobe, occipital lobe, and the posterior cingulate cortex.

**Conclusions:**

The results indicate that in this group of patients, CM was associated with an altered processing of olfactory stimuli, but not development of a functional olfactory deficit. This complements other studies on CM insofar as we found the observed pattern of enhanced activation in associative and emotional regions even following non-traumatic olfactory cues.

## Introduction

Patients with a history of severe childhood maltreatment (CM) seem to have problems with emotional regulation in adulthood [1]. Thus, due to CM, the risk of development of other psychopathology including depression, alcoholism or anxiety disorders increases in later life [2]. Previous research indicates some neuronal correlates of the disease. On a structural level a reduced overall brain volume has been reported [3], [4] in patients with CM, as well as a reduced volume of amygdalae, hippocampus [5], [6] and anterior cingulate cortex [7].

One of the most common disorders associated with CM is Posttraumatic Stress Disorder (PTSD) [8]. Studies using functional imaging, compared responses of participants with and without PTSD while the participants were listening to their personal traumatic reminders. In response to the trauma skripts, PTSD participants showed enhanced activation in amygdalae, orbitofrontal, superior and medial frontal regions [9], [10], [11], [12], [13], the posterior cingulated cortex [8], [14] and in motor areas [8], but reduced activation in the anterior cingulated cortex [10] compared to non-PTSD controls. Nevertheless, heterogeneous results are reported. Lanius therefore hypothesizes different underlying subtypes of traumatic response (for overview see [15]).

It seems to be clear, that patients with CM have an enhanced risk for emotional regulation deficits and might respond to their environment in a different way in adulthood, as increased psychopathology suggests [2]. However, they may also have developed another perception of their environment concomitant with their ‘survival’ of CM. There is some evidence of an altered perception of biographical traumatic memory in CM- participants compared to nontraumatised controls [8], [15], [16]. This seems somewhat predictable given that the biographical memory itself might be much more intense and stressful in CM patients. So using personal memories, the cues are hard to compare. There are many studies indicating, that CM-participants, as well as PTSD-patients, have an altered processing of traumatic cues (see above). But little is known about the processing of daily life stimuli. Thus the aim of the present study was to investigate how women with CM process non-threatening and non-trauma-related stimuli.

We used two edible odors from daily life experience (“peach”; “coffee”) as olfactory stimuli for presentation during in this study. The choice of olfactory stimuli, rather than auditory or visual stimuli, to investigate response to non-trauma-related cues was based on the overlap of brain areas often hypothesized to be altered in CM patients and the projection areas of the olfactory system, like amygdalae, orbitofrontal cortex and hippocampus. Furthermore and in contrast to other sensory systems, much of the olfactory information bypasses the thalamus and projects directly to the amygdalae [17]. Therefore, the sense of smell has a direct link to the affective system [18]. Due to the reported volume reduction and functional peculiarities in parts of the central olfactory processing system in patients with CM and PTSD we expected an altered activation, as detected by fMRI, in these areas in response to olfactory stimuli.

## Materials and Methods

Investigations were performed according to the Declaration of Helsinki on Biomedical Research Involving Human Subjects. The protocol was approved by the University of Dresden Medical Faculty Ethics Review Board and after complete description of the study to the participants, written informed consent was obtained.

### Participants

Twenty-two females participated in the study. All of them were, or have been, patients in the psychosomatic department of the University Hospital Dresden. Twelve of them had a history of CM, 10 reported no CM. There was no significant group difference in age (CM: 38.8+/−11.4y; controls: 41.8+/−9.6y). The CM-group was characterized by suffering more often from PTSD and having a significantly higher severity of PTSD-symptomatology, but did not differ significantly in other mental disorders; especially there were no differences in depression scores between the groups. Characteristics of the two groups are shown in Table 1.

**Table 1:** Demographic variables and questionnaire scores for CM- group and controls.

	Control group (N = 10)		CM group (N = 12)	
	Mean (SD)	Number (%)	Mean (SD)	Number (%)
Age (years)	38.0 (11.4)		41.8 (9.6)	
Smoker. n(%)				
No		6 (62.5%)		9 (75%)
Yes		4 (37.5%)		3 (25%)
Alcohol consumption				
Never. n (%)		2 (25%)		2 (16.7%)
Sometimes		8 (75%)		10 (83.3%)
Regular		0		0
Questionnaire of Depression (BDI)	17.5 (10.2)		18.9 (7.4)	
Questionnaire of Posttraumatic Stress Disorder (IES-R)				
IESR- Intrusion	15.5 (10.2)		22.7 (11.9)	
IESR-Avoidance	21.0 (9.3)		20.6 (11.9)	
IESR- Hyperarousal*	11.6 (8.5)		21.2 (9.1)	
Questionnaire of Childhood Maltreatment (CTQ)				
CTQ – physical abuse *	6.2 (2.63)		13.6 (6.0)	
CTQ – sexual abuse	7.8 (4.9)		12.3 (5.6)	
DIA-X diagnosis				
Substance abuse		1(10%)		0
Depressive disorders		8 (80%)		11 (91.7%)
Anxiety disorders		7 (70%)		8 (66.7%)
Obsessive-compulsive disorder		1 (10%)		2 (16.7%)
Posttraumatic Stress disorder		3 (30%)		6 (50%)
Dissociative disorders		1 (10%)		1 (8.3%)
Somatoform disorders		6 (60%)		7 (58.3%)
Eating disorders		1 (10%)		1 (8.3%)
Sum of DIA-X diagnosis per patient	3.7 (1.7)		3.3 (2.1)	

*Significant difference between CM-group and controls (p<0.05).

Enrollment of the participants took place in several steps. From all patients of the psychosomatic clinic we included women without severe neurological diseases, like epilepsy. Furthermore, we excluded patients with chronic or acute nasal diseases, because this might affect odor perception. We then analyzed psychotherapeutic interviews performed with the patients. These interviews were performed by a psychotherapist and were part of the normal treatment in the psychosomatic clinic. Patients were eligible for inclusion into the CM-group only if they explicitly spoke about their own experience of CM in this interview and, additionally, if they scored higher than 11 on the Childhood Trauma Questionnaire subscales “sexual abuse” or “physical abuse” [19], [20]. Patients were recruited for the control group if there were was no evidence of possible CM in the psychotherapeutic interview. All of the patients suitable for the CM and control group were invited to participate. About 70% of women eligible for the CM or control group agreed to participate in the study.

Participants with CM reported significantly more physical (p = 0.003) and often more sexual abuse (p = 0.06) than participants of the control group. Compared with a USAmerican- normative sample [21], participants of the CM-group scored significantly above the mean in both subscales (sexual abuse p = 0.002; physical abuse p = 0.002). The controls did not score significantly different from this normative sample.

All of the participants underwent a standardized interview for diagnosis of mental disorders [22] based on the American Psychiatric Association's Diagnostic and Statistical Manual of Mental Disorders (DSM-IIIR). In both groups, most of the participants fulfilled criteria for depressive disorders, somatoform disorders or anxiety disorders (see Table 1). Groups did not differ significantly according to the diagnosis of mental disorders; neither for specific diagnosis nor for the sum of the diagnosis. Groups also did not differ in the self-reported severity of depressive symptoms, measured via Becks Depression Inventory (BDI [23], [24]. However, there was a significant difference in the self-reported severity of PTSD-symptomatology, measured with a questionnaire for PTSD-Symptoms (IES-R - Impact of Event Scale - Revised [25], [26]. Data collected using this questionnaire showed that there was no significant difference in the subscales “avoidance” and “intrusion”, but that the CM-group reported significantly higher scores in the “hyperarousal”-subscale (p = 0.02). Using the “Sniffin' Sticks” test kit [27], [28] normal olfactory function was present in all participants.

### Stimulus Presentation

We used a three-factorial design with the between subject factor, “group” (CM vs. controls), and two within subject factors, “side” and “odor”, where side refers to the nostril tested (left/right) and odor refers to the odor presented (coffee/peach). Each subject participated in four sessions with two very common ‘food-like’ olfactory stimuli (peach and coffee-like odors) presented in randomized order unilaterally to the right and then the left nostril. Odors were presented intranasally (inner diameter of the Teflon™ tubing 4 mm). To avoid mechanical stimulation the odor pulses were embedded in a constant flow of odorless, humidified air. Stimulus pulses had a duration of 1 s, the interval between stimuli was 2 s. After each session participants rated intensity (0 = extremely low intensity; 10 = extremely high intensity) and hedonic quality (−5 = extremely unpleasant; +5 = extremely pleasant) of the odors.

### FMRI Protocol and Data Analysis

We used a 1.5T scanner (SONATA-MR; Siemens, Erlangen, Germany) for FMRI data acquisition. For functional data 96 volumes per session were acquired by means of a 26 axial-slice matrix 2D SE/EP sequence (TR:2630ms/TE:45ms, matrix = 64×64, voxel size 3×3×3,75mm^3^). Sessions were randomized across participants. In each session the participants received 8 scans during the 20s ON-block and 8 during the 20s-OFF-block. ON and OFF blocks were repeated 6 times, each session lasted 4 min. Additionally, T1-weighted images were acquired by using a 3D IR/GR sequence (TR: 2180ms/TE: 3.39ms) to localize the activated areas.

Data analysis was performed with SPM 5 software (Statistical Parametric Mapping; Wellcome Department of Imaging Neuroscience, in the Institute of Neurology at University College London [UCL], UK), implemented in Matlab R2007b (Math Works Inc., Natick, MA, USA), following spatial pre-processing with the same software (spatial filtering: high pass filter 128Hz, normalisation using segmentation procedure, smoothing by means of 6×6×6 FWHM). Coordinates of the activation are presented according to Talairach [29]. Analysis was based on t-test with cluster level of 6, and p<0.001 (uncorrected). In order to test our hypothesis of altered activation in olfactory processing areas, we performed a Small Volume Correction using Masks for primary (amygdala; piriform cortex) and secondary (orbitofrontal cortex; insula; hippocampus; thalamus) olfactory areas. Masks were created using the WFU PickAtlas 2.4 software [30].

## Results

### Odor Threshold and Identification Results

There was no significant difference according to the psychophysiological odor performance between the groups (CM-group: odor threshold mean 7.1 (standard deviation [SD] 2.3) odor identification mean 25.0 (SD 7.0) controls: odor threshold mean 6.7 (SD 2.9) odor identification mean 22.5 (SD 7.0)).

### Odor Ratings

**Figure 1:**
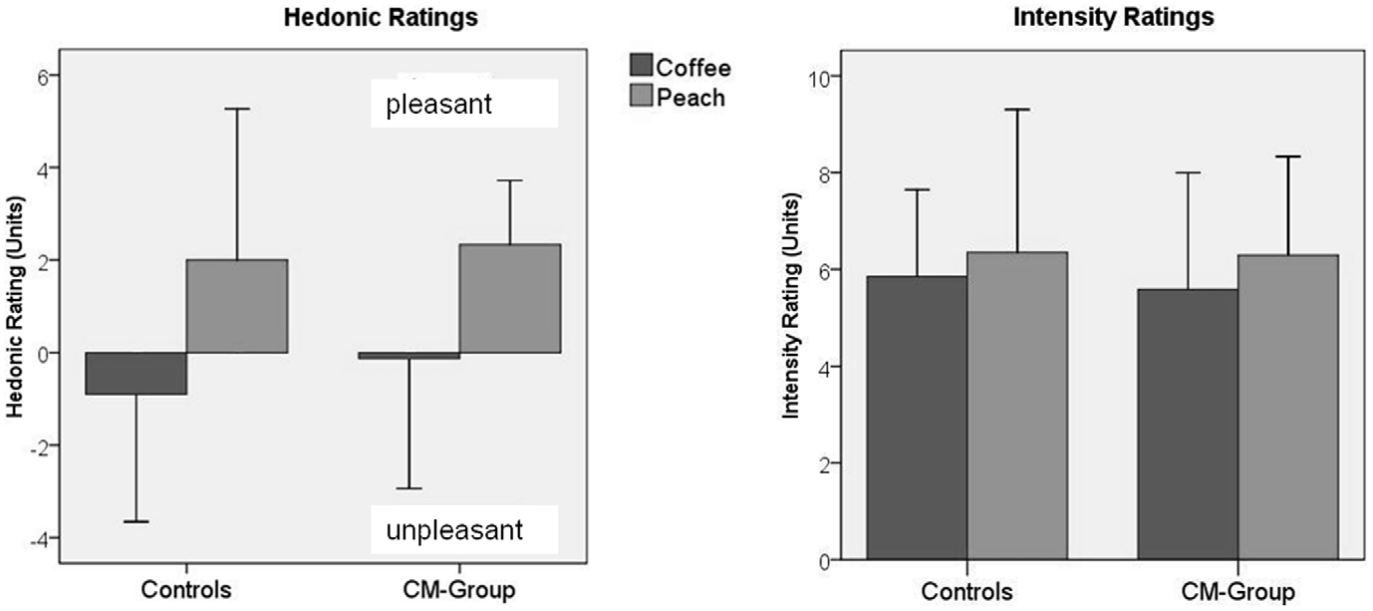
Hedonic and intensity ratings (means, standard deviations [SD]) for peach-odor and the coffee-like odor in CM- and control group. Higher ratings mean greater perceived pleasantness and/or intensity. For convenience, standard deviations are presented one-sided.

There was no apparent difference between the judged intensity of the two odors (p = 0.16), but their hedonic quality was rated differently by both groups (p<0.001, see Figure 1). While “peach” was judged to be very pleasant, the “coffee-like” odor was rated to be neutral. There were no differences in the perceived intensity or hedonic judgments of these odors between the CM and the Control group.

### Main Effect of “Odor”

First, we analyzed the main contrast of ON(odor)- vs. OFF-conditions separately for both groups focusing on the aspect of olfactory processing. In both groups we found significantly enhanced activation in areas that are typically involved in the processing of olfactory stimuli. This included primary and secondary olfactory areas like amygdalae, insula and orbitofrontal cortex (for details see Table 2).

**Table 2:** Odor – No Odor Contrast in CM-group (N = 12) and controls (N = 10); significant peaks of activation in the small volume corrected areas are presented (cluster level 6, p_uncorr_<0.001).

CM-group Odor vs No Odor						
				TAL coordinates		
		cluster size	t-value	X	Y	Z
Primary olfactory areas	Amygdala (left)	61	4.29	−20	−5	−13
Secondary olfactory areas	Orbitofrontal Cortex (right)	72	5.68	34	27	−6
	Orbitofrontal Cortex (left)	192	4.97	−48	21	−6
		30	4.17	−22	25	−15
		11	3.68	−38	34	−13
		7	3.56	−24	33	−8
	Insula (right)	98	4.63	34	27	−5
	Insula (left)	118	5.32	−38	1	−10
		21	3.97	−42	17	−3
		12	3.63	−30	18	3
	Hippocampus (left)	28	4.23	−16	−12	−15
**Controls Odor vs No Odor**						
Primary olfactory areas	Amygdala (left)	19	3.97	−18	−5	−15
Secondary olfactory areas	Orbitofrontal Cortex (left)	88	4.25	−20	27	−13
		15	4.07	50	29	−5
		7	3.50	−42	15	−6
	Orbitofrontal Cortex (right)	21	4.03	24	28	−13
	Insula (left)	34	4.45	−34	−7	13
		90	4.42	−30	19	1
		9	3.46	−42	13	−6

### Comparison between the Groups for Both Odors

We compared the ON-contrasts of the CM-group with the ON-contrasts of controls and performed the same Small Volume Correction for olfactory processing areas described above. This contrast revealed no suprathreshold activations of olfactory processing areas in the CM-group compared to controls. However, for controls we found increased activation in the left hippocampus (−30/−28/−10; t = 3.96; cluster size 12) and in the left orbitofrontal cortex (−10/48/−9; t = 4.36; cluster size 29) compared to the CM-group.

**Figure 2:**
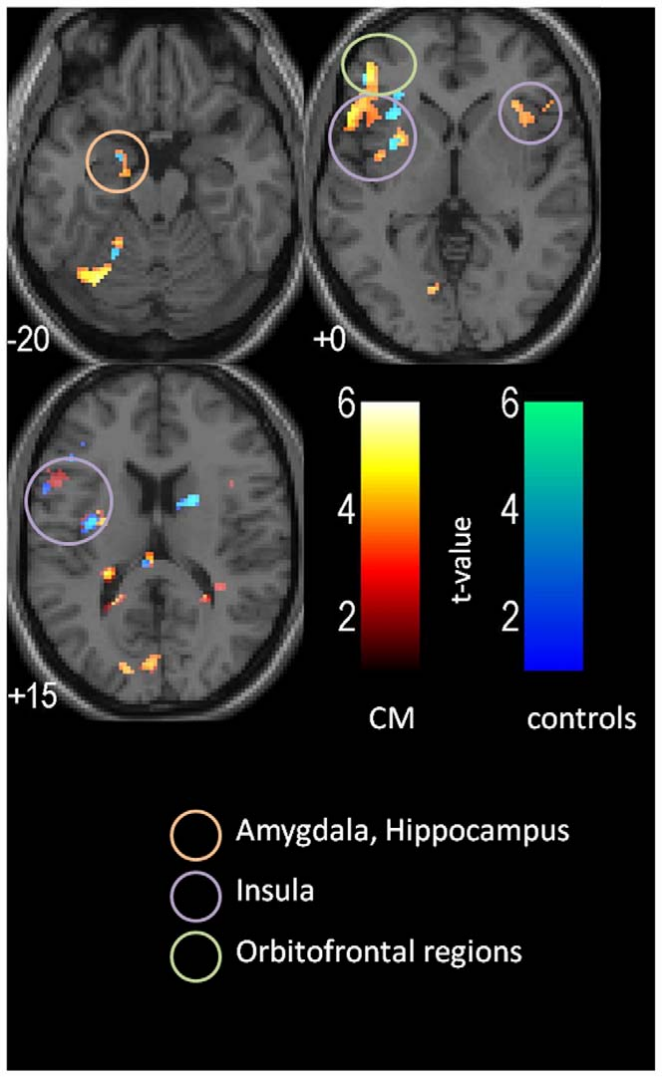
Activated clusters (k≥6; p≤0.001) for both odors in the CM group (orange) and in the controls (blue) (contrasts: ON vs. OFF separately for the two groups). For visualization, we used a normalized template, provided by SPM 5 –Software (single_subj_T1.nii).

**Figure 3:**
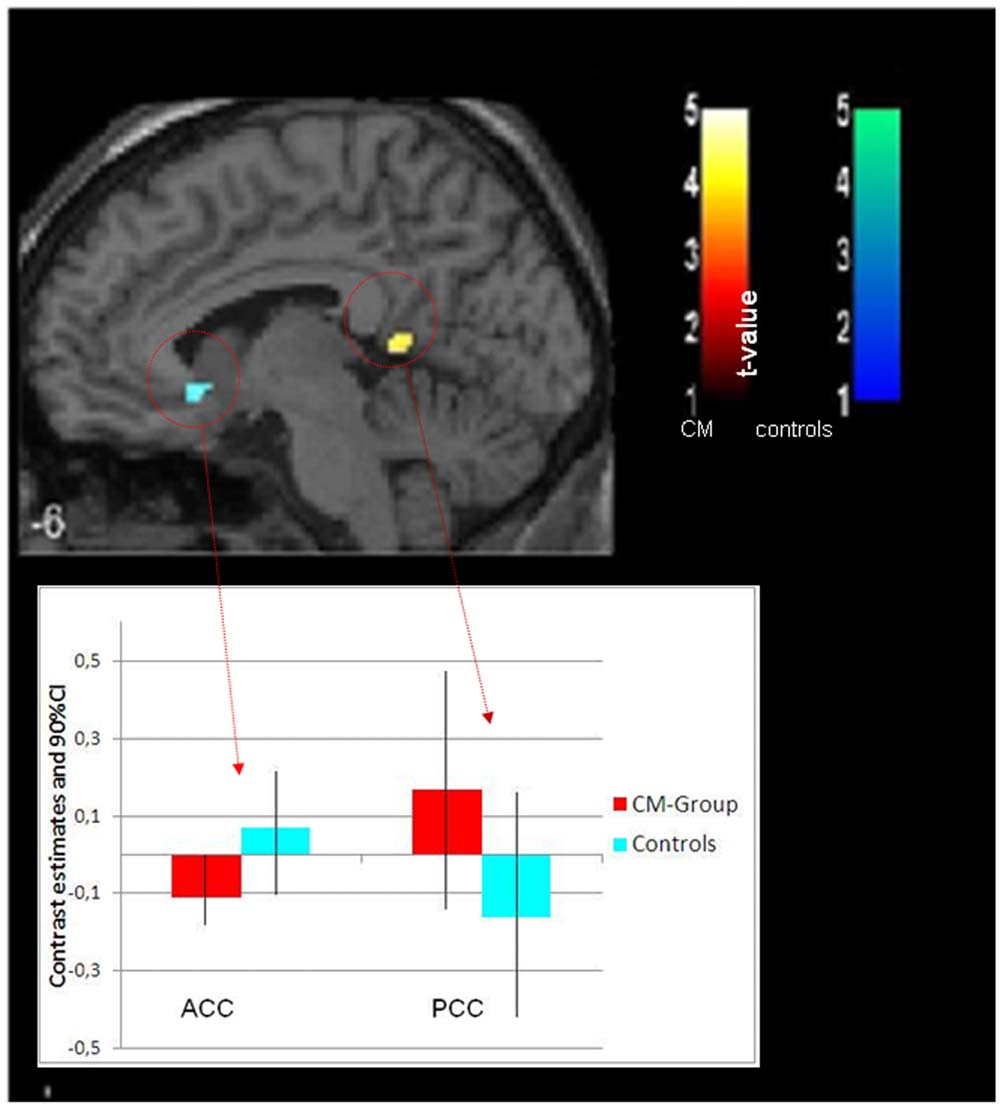
Activated clusters for contrast: CM group vs. control group in the x = −6 slice (k≥6; p≤0.001) for both odors. The CM group (orange) exhibits activation in the posterior cingulate cortex (PCC) while control subjects (blue) show activation in the anterior cingulate cortex (ACC). For visualization, we used a normalized template, provided by SPM 5 –Software (single_subj_T1.nii).

We performed a whole brain analysis, to look for activation differences in response to the olfactory stimuli other than those in the olfactory processing areas. The contrast CM-group vs. controls revealed significantly enhanced activation in the CM-group in various areas compared to the controls. These areas include neocortical regions in the middle, inferior and precentral frontal gyrus, middle temporal gyrus, inferior, and supramarginal parietal areas, in the occipital cortex and cerebellum, as well as in the posterior cingulate cortex, a part of the limbic system. The reverse, controls vs. CM-group contrast, indicated significant enhanced activations in the cerebellum and in the anterior cingulate cortex (for details see Table 3 and Figures 2 and 3).

**Table 3:** Comparison of the activation in groups with data pooled odors; significant peaks of activation are small volume corrected for the olfactory processing areas and without correction for other areas (whole brain analysis); cluster level 6, p_uncorr_<0.001.

CM group vs. Control group						
				TAL coordinates		
		cluster size	t-value	X	Y	Z
Frontal Lobe*	Inferior Frontal Gyrus	22	4.29	44	5	31
	Precentral Gyrus	14	3.72	−42	0	30
		12	3.70	−51	−18	36
	Middle Frontal Gyrus	6	3.61	36	22	19
Temporal Lobe*	Middle Temporal Gyrus	14	4.28	−53	−35	−8
		10	3.85	−61	−31	−2
Parietal Lobe*	Inferior Parietal Lobule	19	3.79	46	−41	43
	Supramarginal Gyrus	7	3.78	−51	−39	33
Occipital Lobe*	Cuneus	46	4.01	−12	−75	11
	Lingual Gyrus		3.88	−12	−72	4
	Cuneus	8	3.40	−14	−84	30
Limbic Lobe*	Posterior Cingulate	39	3.96	−4	−42	9
Cerebellum*	Declive	14	3.69	−38	−63	−14
Primary olfactory areas**		-no suprathreshold voxels-				
Secondary olfactory areas**		-no suprathreshold voxels-				
**Controls vs. CM group**						
Frontal Lobe*	Orbitofrontal	35	4.48	−12	46	−9
Limbic system*	Hippocampus	7	3.51	−30	−28	−10
	Anterior Cingulate	40	4.70	−8	19	−8
			3.46	−12	10	−4
Cerebellum*	Culmen	11	3.54	−24	−40	−15
Primary olfactory areas**		-no suprathreshold voxels-				
Secondary olfactory areas**	Orbitofrontal Cortex (left)	29	4.36	−10	−48	−9
	Hippocampus (left)	12	3.78	−30	−28	−10

*whole brain analysis.

**Small Volume Correction for the defined masks.

### Comparison between the Groups during Coffee Odor Presentation

Comparison of the ON-contrasts of the CM-group with the ON-contrasts of controls revealed no suprathreshold voxels in the primary or secondary olfactory processing areas (using the Small Volume Correction, described above). However, the CM-group showed significantly increased activation compared to the controls in inferior, paracentral and middle frontal and middle temporal regions, in postcentral, inferior and supramarginal parietal regions, in the occipital lobe, the posterior cingulate cortex and in the lentiform nucleus (whole brain analysis). The reverse, controls vs. CM-group contrast, revealed increased activation in the orbitofrontal cortex (small volume corrected). In the whole brain analysis we found additionally enhanced activation in the temporal lobe and in the caudate compared to the CM-group (for details see Table 4).

**Table 4:** Comparison of the activation data from groups for each odor separately; significant peaks of activation are small volume corrected for the olfactory processing areas and without correction for other areas (whole brain analysis); cluster level 6, p_uncorr_<0.001.

Coffee-like odor: CM group vs. Controls						
				TAL coordinates		
		cluster size	t-value	x	y	Z
Frontal Lobe*	Inferior Frontal Gyrus	22	4.60	44	5	31
	Paracentral Lobule	6	3.77	−6	−29	47
	Middle Frontal Gyrus	11	4.24	50	29	26
	Sub-Gyral	21	3.97	32	45	1
Temporal Lobe*	Middle Temporal Gyrus	93	4.81	−63	−39	4
	Middle Temporal Gyrus		4.62	−61	−31	−2
	Middle Temporal Gyrus	12	4.59	−53	−35	−8
Parietal Lobe*	Postcentral Gyrus	14	4.05	−55	−18	23
	Inferior Parietal Lobule	16	3.90	46	−39	41
	Supramarginal Gyrus	6	3.67	50	−43	33
Occipital Lobe*	Cuneus	7	3.62	−14	−77	11
Limbic Lobe*	Posterior Cingulate	45	4.40	−8	−46	12
Sub-lobar*	Lentiform Nucleus	22	4.29	24	12	7
	Lentiform Nucleus	8	3.52	−26	−19	−1
Primary olfactory areas**		-no suprathreshold voxels-				
Secondary olfactory areas**		-no suprathreshold voxels-				
**Coffeelike odor Controls vs. CM group**						
Frontal Lobe*	Orbitofrontal	23	3.94	−12	46	−7
Temporal lobe*	Extra-Nuclear	11	3.77	−16	−50	19
Sub-lobar*	Caudate	18	4.14	−10	13	−4
Primary olfactory areas**		-no suprathreshold voxels-				
Secondary olfactory areas**	Orbitofrontal Cortex (left)	14	3.94	−12	46	−7
**Peach CM group vs. Controls**						
Frontal Lobe*	Sub-Gyral	14	3.91	−22	−24	29
Parietal Lobe*	Supramarginal Gyrus	9	3.76	−51	−39	33
	Sub-Gyral	7	3.57	30	−41	41
Limbic System*	Cingulate Gyrus	6	3.71	14	−24	31
	Cingulate Gyrus	17	3.52	−18	7	33
Primary olfactory areas**		-no suprathreshold voxels-				
Secondary olfactory areas**		-no suprathreshold voxels-				
**Peach Controls vs. CM group**						
		No suprathreshold voxels				

*whole brain analysis.

**Small Volume Correction for the defined masks.

### Comparison between the Groups for the Peach Odor

The comparison of the ON-contrasts of the CM-group with the ON-contrasts of controls again revealed no suprathreshold voxels in the primary or secondary olfactory processing areas (small volume corrected). In the whole brain analysis there was significantly increased activation in the cingulate and the supramarginal parietal cortex of CM participants compared to the controls. There were no significant suprathreshold clusters in the control vs. CM-group contrast for peach odor (for details see Table 4).

## Discussion

Following presentation of relatively neutral up to pleasant rated olfactory stimuli, neural activation was observed in the primary and secondary olfactory systems, including amygdalae, insula and orbitofrontal cortex [17]. As expected, these activations were present in both groups. So both, women with CM as well as control participants showed normal activation in the olfactory processing areas. However, the CM vs. controls contrast yielded enhanced activation in multiple, mainly neocortical, regions involved in association networks. Additionally the CM-group showed altered activation in limbic areas, including enhanced activation in the posterior cingulate cortex and decreased activation in the anterior cingulate cortex. This seems to support the hypothesis of an altered processing of non-traumatic stimuli in CM patients. The effect seems more pronounced following stimulation with the neutral coffee-like odor, than following stimulation by the pleasant peach odor.

***Increased activation in the CM-Group*** compared to the controls was spread widely over various neocortical areas and in the posterior cingulate cortex, as part of the limbic system. Specifically, increased activation was found in the precentral frontal lobe, which has a strong association with motor processing and in inferior and middle frontal structures, involved in speech production [31]. Activation of the middle temporal gyrus is related to episodic and semantic memory processes, as well as with language [32] and multi-modal sensory integration [33]. The posterior parietal lobe is part of the association cortex and involved in spatial awareness [33]. The occipital lobe has strong relationships with the visual system, while the posterior cingulate cortex is assumed to be involved in modulation of emotions [34].

In addition to the increased activation in various regions, analysis revealed ***decreased activation in the CM-Group*** in anterior cingulate regions compared to the controls. The anterior cingulate cortex is thought to be involved in the emotional processing [35] as well as in attention [36] and working memory [15], [37]. This pattern could potentially be understood as an enhanced activation of different sensory and motoric systems and an altered activation of emotional systems in the CM-group compared to controls following presentation of olfactory stimuli.

When focusing on the primary and secondary olfactory areas, we found no group differences in the activation of ***primary olfactory areas***. However, we found ***decreased activation in the secondary olfactory processing areas*** of hippocampus und orbitofrontal cortex in the CM-group compared to controls. This effect vanishes when analyzing the two odors separately, very likely because of the reduced statistical power. Therefore, only the decreased activation in orbitofrontal regions in the CM-group compared to the controls for the relatively neutral coffee-like odor remains stabile. The orbitofrontal cortex is involved in working memory [38], emotional regulation [39], as well as in odor memory, odor identification and discrimination abilities [40], [41], [42], [43]. One could hypothesize, that the CM-group did worse in secondary cognitive processing of the odor, but the results of the Sniffin'Sticks testing showed no group differences and the effect seems not very stable.

Another, recently published, imaging study dealt with odors in traumatized combat veterans [44]. The authors used diesel odor as a very traumatic reminder of affective episodes for war veterans. This study revealed similar increased posterior cingulate activations in those veterans suffering from PTSD. Additionally, PTSD-participants showed enhanced amygdalae activation. In our study, we did not operate with traumatic odors, but with a neutral and a pleasant one, as the hedonic judgments of the odors suggest. Furthermore, no participant reported any flashback or dissociative states during the scanning. Therefore, it seems reasonable that we did not replicate significant amygdala activation compared to the controls. Nevertheless, we see the very same increase in posterior cingulate regions in traumatized patients following olfactory stimuli. This suggests ongoing and generalized alteration in olfactory stimuli processing in the posterior cingulate brain regions in people with a history of traumatic experience.

Our results are in accord with other FMRI studies dealing with aversive auditory cues in traumatized patients [8], [15], [16], [45]. Two studies, using a traumatic memory recall paradigm, yielded in highly comparable patterns of enhanced activation in motor, parietal, visual and posterior cingulate areas and reduced activation in anterior cingulate areas has been found [15], [16]. Indeed it is rather surprising that we found similar enhanced activations in traumatized patients without using auditory traumatic stumuli, but rather neutral to pleasant olfactory stimuli.

A positron emission tomography (PET) study using traumatic memory recall paradigm, compared responses of patients with childhood sexual abuse related PTSD with those from participants with childhood sexual abuse without PTSD [8]. In this study, the PTSD-affected participants demonstrated increased activation in superior and middle frontal, temporal, precentral and posterior cingulate structures and no activation in the anterior cingulate cortex compared to non-PTSD affected participants. The authors state that these areas might be functionally linked and operate together in the mediation of traumatic remembrance in PTSD patients. In an fMI study, Lanius and colleagues asked participants to recall different aversive emotional states. They found decreased anterior cingulate activation in the PTSD-group compared to participants, who were also traumatized, but did not suffer from PTSD [45]. In those studies, alterations in brain activity appear not to be mediated by the experience of trauma, but rather by PTSD-symptomatology.

Our groups also differed in PSTD-severity as described above, although initially grouped for CM-experience. Participants of the CM-group reported significantly higher PTSD-hyperarousal scores than did the controls. It is a considerable limitation of our study, that both groups are non-comparable with regard to the PTSD severity. Although it might reflect reality, that most patients with CM have higher PTSD-scores, the internal validity of our study is constricted. Further research is necessary to ascertain, if the effect of altered processing of nontraumatic olfactory stimuli in CM patients is due to current psychopathology or due to the individual biographical experience.

Another design limitation of the study is the relatively small sample size. To further validate the results, similar studies with an increased sample size are needed. Additionally the study focuses on women. Comparison of our results with the olfactory imaging study on male combat veterans, mentioned above, shows increased similar posterior cingulate activation. Still we cannot generalize our results to men.

As we found very few differences between the research groups in olfactory processing areas, we would argue that the pattern of enhanced activation in emotional and associative areas in CM participants should not be specific to olfaction. Further research should explore whether CM participants also show a similar patterns of enhanced and reduced activation following exposure to non-traumatic auditory or visual cues.

To our knowledge, no previous study has shown that a group of patients with psychosomatic disorders and CM exhibit altered processing of nontraumatic olfactory stimuli. The pattern of enhanced activation of different sensory and motoric systems suggest that these women focus less passively on the stimuli, but immediately connect to associative functions, like speech, other sensory systems or to motoric function. This might be accompanied by altered perception of the stimuli itself or of its environment, but further research on this topic is necessary. The present findings also underline the usefulness of olfactory probes in the investigation of certain brain pathologies.
